# Screen-Printed Sensor for Low-Cost Chloride Analysis in Sweat for Rapid Diagnosis and Monitoring of Cystic Fibrosis

**DOI:** 10.3390/bios10090123

**Published:** 2020-09-11

**Authors:** Alicia Hauke, Susanne Oertel, Leona Knoke, Vanessa Fein, Christoph Maier, Folke Brinkmann, Michael P. M. Jank

**Affiliations:** 1 Fraunhofer Institute for Integrated Systems and Device Technology, 91058 Erlangen, Germany; susanne.oertel@iisb.fraunhofer.de (S.O.); michael.jank@iisb.fraunhofer.de (M.P.M.J.); 2 Department of Paediatric Pneumology, University Children’s Hospital, Ruhr-University Bochum, 44791 Bochum, Germany; leona.knoke@rub.de (L.K.); vanessa.fein@rub.de (V.F.); christoph.maier@rub.de (C.M.); folke.brinkmann@rub.de (F.B.)

**Keywords:** screen-printed sensor, sweat analysis, ion-selective electrode, chloride, cystic fibrosis

## Abstract

Analysis of sweat chloride levels in cystic fibrosis (CF) patients is essential not only for diagnosis but also for the monitoring of therapeutic responses to new drugs, such as cystic fibrosis transmembrane conductance regulator (CFTR) modulators and potentiators. Using iontophoresis as the gold standard can cause complications like burns, is uncomfortable, and requires repetitive hospital visits, which can be particularly problematic during a pandemic, where distancing and hygiene requirements are increased; therefore, it is necessary to develop fast and simple measures for the diagnosis and monitoring of CF. A screen-printed, low-cost chloride sensor was developed to remotely monitor CF patients. Using potentiometric measurements, the performance of the sensor was tested. It showed good sensitivity and a detection limit of 2.7 × 10^−5^ mol/L, which covered more than the complete concentration range of interest for CF diagnosis. Due to its fast response of 30 s, it competes well with standard sensor systems. It also offers significantly reduced costs and can be used as a portable device. The analysis of real sweat samples from healthy subjects, as well as CF patients, demonstrates a proper distinction using the screen-printed sensor. This approach presents an attractive remote measurement alternative for fast, simple, and low-cost CF diagnosis and monitoring

## 1. Introduction

Sweat analysis is an emerging field that provides insights into human health, and over the last few years has become an attractive, non-invasive alternative to blood analysis [1,2,3,4,5]. Sweat analysis is more comfortable for patients, has good accessibility, and is able to continuously monitor body parameters, which makes sweat analysis useful for monitoring health and fitness [4].

The development of so-called wearable devices increases rapidly due to new techniques of miniaturization, flexibility, and low-cost production. The number of publications in the field of sweat analysis rises steadily every year. Most of them use electrochemical sensors, especially ion-selective electrodes (ISEs), to analyze specific ions in sweat. Due to new developments in printing paste technologies, it is possible to produce screen-printed, ion-selective electrodes on flexible polymeric substrates, which are also inexpensive, due to mass fabrication [6,7,8]. Thus, screen-printing technology is preferred for the production of disposable, low-cost sweat sensors with small dimensions, in contrast to commonly used glass or tube ion-selective electrodes, which need high maintenance, high sample volume, and are rigid and expensive.

Sweat sensors integrating ion-selective electrodes can be used to collect vital data parameters like fatigue or fluid balance during sports, and can also support or replace diagnostic or monitoring measures. In addition to water, sweat contains characteristic plasma-related electrolytes, namely sodium (Na^+^), potassium (K^+^), and chloride (Cl^−^) [1,9]. For example, the sodium concentration is linked to dehydration and electrolyte imbalance [10,11,12], whereas the potassium concentration in sweat is related to hypo-/hyperkalemia [13,14].

The detection of cystic fibrosis (CF) is one of the most used sweat diagnostics [15,16]. CF is a genetic disease, which causes a defective ion transfer through the epithelial cellular membranes (cystic fibrosis transmembrane conductance regulator (CFTR) chloride channels). As a result, chloride reabsorption from sweat is reduced, leading to a sticky mucus formation, which, if untreated, can be fatal. Therefore, an early diagnosis is crucial for symptomatic treatment to improve the quality of life and life expectancy of patients. As the gold standard for CF diagnosis, chloride concentration is determined using sweat tests [16,17]. In addition to this, a new generation of CF drugs, called CFTR modulators and potentiators, changes the chloride current across the epithelial barrier, and therefore, the chloride concentration in the sweat, which can be used as therapeutic monitoring.

During these tests, sweating is induced by so-called pilocarpine iontophoresis. In this procedure, a low current is applied between two electrodes attached to the forearm of the patient. For sweat inducement, a solution of pilocarpine is applied under the anode. Through the low-voltage induced electric current, pilocarpine is carried into the skin, where it stimulates the sweat glands. The sweat is then collected and analyzed regarding its chloride concentration.

Especially in terms of the current COVID-19 pandemic, the risk of infection for risk groups has increased, including CF-patients. Therefore, there is a high demand for an easy outpatient approach for sweat chloride measurement. The chloride concentration can be acquired by using a screen-printed chloride sensor, which can be used as a portable device and is user-friendly.

Beyond this, the use of portable sensors can help increase our understanding of CF. Due to the time-intensive setting of pilocarpine iontophoresis, which can only be carried out under medical supervision in clinics, the procedure is usually conducted on the patients in intervals of several months. As a result, there is almost no understanding of the development of the chloride concentration in the everyday life of the patient, and how it is affected by factors like CFTR modulators and other drugs, physical activity, rest, or sleep. A new, portable sweat sensor would allow patients to measure the chloride concentration in their sweat more regularly and, in doing so, it would provide data that would be useful for understanding the CF disease.

Within our study, a screen-printed chloride sensor was developed, and its performance was tested to show its usability as a CF sensor. The sensor was tested for its sensitivity, detection limit, and response time. As the sensor achieved the necessary performances under laboratory conditions, real sweat samples were measured for the determination of chloride concentrations, to validate the correct detection of CF. Sweat samples of CF patients were collected under stable clinical conditions, in the absence of acute infections.

## 2. Materials and Methods

### 2.1. Sensor Fabrication

**Figure 1:**
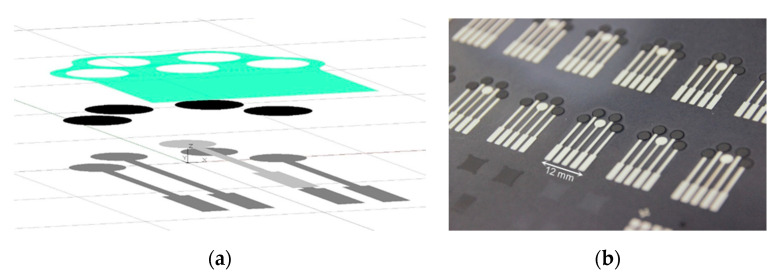
(**a**) Explosion view of screen-printed chloride sensor depicting the layers. (i) Silver (dark grey), (ii) silver/silver chloride (light grey), (iii) carbon (black), and (iv) insulation/encapsulation (green). (**b**) Manufactured sample sheet of sensors before deposition of the selective and reference membranes.

For the analysis of sweat samples, a screen-printed potentiometric chloride sensor was developed. Sensor electrodes were fabricated using a semiautomatic screen and pattern printer from Ekra (series XH STS). Each electrode, consisting of different layers (see Figure 1a), was printed using a polyester screen. Between each printing step, the layer was annealed at 120 °C to 130 °C, for 3 min and up to 10 min, depending on the used printing paste. PET foils were used as substrates to yield a flexible sensor that could later be attached to the skin (see Figure 1b). The working electrode of the potentiometric sensor was printed using a silver paste as a conducting layer, covered by a carbon layer as solid contact. Additionally, a reference electrode was printed using a silver/silver chloride paste. For encapsulation of the electrodes, an insulating layer was printed on top of both electrodes, leaving the active parts of the working and the reference electrodes open. All printing pastes were purchased from Du Pont (UK) Limited.

To functionalize the electrodes, an ion-selective membrane was drop-casted on top of the working electrode. It consisted of a chloride ionophore I - cocktail A from Sigma Aldrich, mixed with polyvinyl chloride as polymer and tetrahydrofuran as solvent. The resulting composition of the membrane is listed in Table 1. To achieve a constant potential of the reference electrode, a membrane consisting of polyvinyl butyral, methanol, and sodium chloride was used. The procedure was already described in previous studies [18,19].

### 2.2. Potentiometric Measurements

The performance of the fabricated sensors was tested using potentiometry; a high resolution, high-input impedance, multi-channel potentiometer (EMF 16, Lawson Labs, Malvern, PA, USA) was utilized. The functionality of the sensors was validated in solutions of NaCl and NH_4_Cl with increasing concentrations from 10^−6^ mol/L to 10^−1^ mol/L measuring the potential of the ion-selective electrode versus the reference electrode. For the measurement of sweat samples, the calibration range was extended until 1 mol/L. The potential response of the ISEs was recorded for a representative response of Cl^−^. The resulting calibration plot was used to determine the limit of detection and the slope of the presented electrodes, as well as the response time.

### 2.3. Sweat Inducement and Collection

**Figure 2:**
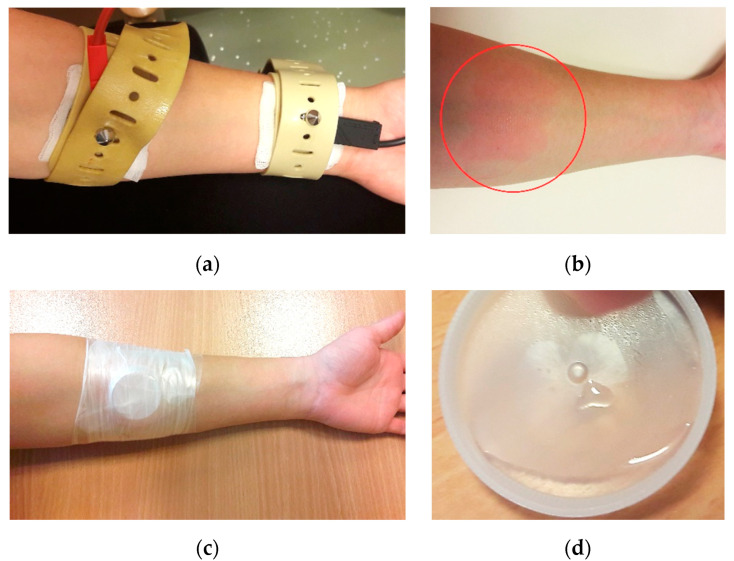
(**a**) Sweat inducement by pilocarpine iontophoresis using two electrodes attached to the forearm; (**b**) irritation of skin after iontophoresis due to the applied electric current; (**c**) test tube pressed onto the forearm in the former position of the pilocarpine electrode to collect sweat; and (**d**) small amount of collected sweat with test tube after pilocarpine iontophoresis.

For the withdrawal of sweat and analysis of the chloride concentrations, we applied the most commonly used method of pilocarpine iontophoresis. Sweat excretion was induced by a small current between two electrodes attached to the forearm of the test subject, which drove pilocarpine into the epidermis (Figure 2a). After five minutes, the electrodes were removed (Figure 2b) and a test tube was pressed onto the forearm, in the former position of the pilocarpine electrode, to collect sweat (Figure 2c,d). The collection time took 20 min; thereafter, the chloride concentration of the sweat samples was measured using a Kreienbaum FKGO Chloridmeter, which is the established method in CF diagnosis at the University Children’s Hospital, Ruhr-University Bochum. The remaining sweat was frozen at −20 °C and stored until measurements were taken using the printed chloride sensor. To collect a sufficient amount of sweat for both methods, iontophoresis was conducted on both forearms, after which the samples were combined.

We included patients with genetically confirmed CF, alongside healthy patients, as test subjects for this pilot study. All tests were carried out under professional medical monitoring. All human trial regulations were met.

## 3. Results and Discussion

### 3.1. Sensor Performance

Screen-printing technology facilitates the production of large-scale reproducible sensor electrodes. However, the functionalization of the electrodes through drop-casting of membranes creates slight deviations between the ion-selective electrodes. Therefore, it is crucial to calibrate each sensor and characterize its performance regarding its sensitivity, the limit of detection, the linear range, and its response time.

**Figure 3:**
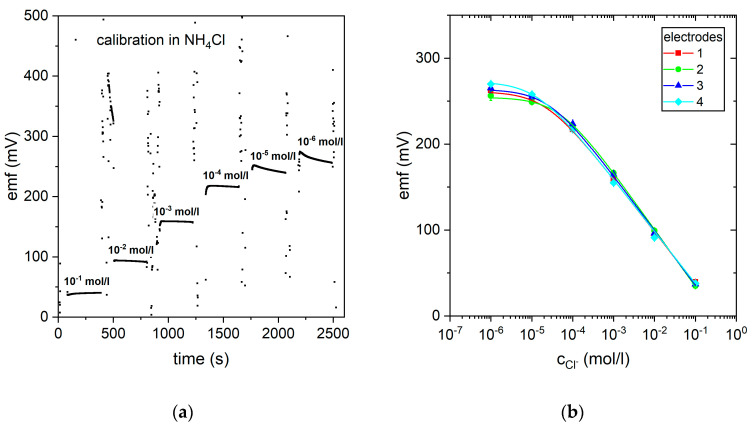
(**a**) Potentiometric response curve of the screen-printed chloride sensor measured in NH_4_Cl solutions with different concentrations. (**b**) Resulting calibration plot measured in NH_4_Cl solutions of four different chloride-selective electrodes sharing one reference electrode.

To apply the chloride sensor for the initial diagnosis of CF, it is mandatory that the sensor covers the possible concentration range of chloride in sweat (typically 1 mM to 200 mM) and has a reasonable response time. Figure 3a shows a typical potentiometric response curve for a chloride sensor in NH_4_Cl standard solution with varying concentrations. As depicted, the sensor reaches a stable potential over time, for concentrations above 10^−5^ mol/L. The evaluated response time in this region was as low as 30 s. Below 10^−5^ mol/L, the potential decreased continuously after reaching an initial peak indicative of the actual concentration. However, this was not crucial for the application in CF diagnostics, as the lowest possible concentration of chloride in sweat only reached about 10^−3^ mol/L [1].

Calculating the mean value of each potentiometric step after reaching a constant potential and using the Nernstian equation [20], the calibration plot could be fitted. Figure 3b shows the calibration plot of four different chloride ISEs sharing one reference electrode in the NH_4_Cl solution, utilizing the sensor layout shown in Figure 1. Figure 3b shows that all four electrodes achieved a reproducible dependence on the chloride concentration with a sensitivity of 62.9 mV/dec. This follows the ideal Nernstian slope of 59.6 mV. Using the fitted calibration plot, a detection limit of 2.7 × 10^−5^ mol/L and a linear behavior between 10^−4^ mol/L and 10^−1^ mol/L could be determined.

These results showed that the sensor had excellent performance, covering the complete concentration range of chloride in sweat, showing fast response time and good potential stability, meaning that this sensor was highly applicable. As the sensor could be produced at a low cost, and was small and flexible, it could be used as a portable device. This sensor had significant advantages over comparable methods. A comparison to the standard systems used in clinical diagnostics is listed in Table 2.

### 3.2. Measurement of Sweat Samples

**Figure 4:**
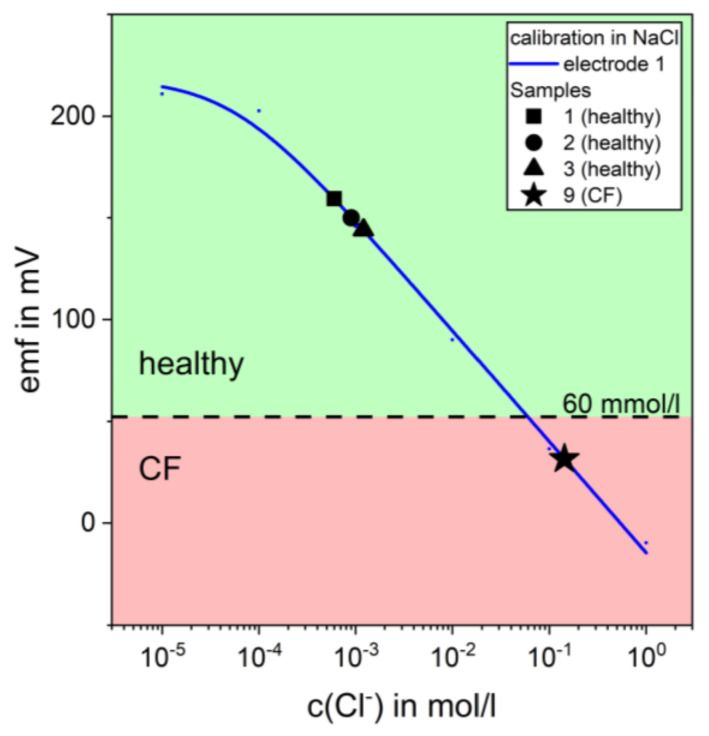
Measurement of sweat samples (samples 1, 2, 3, and 9) using the screen-printed chloride sensor for the detection of cystic fibrosis (CF). The dashed line displays the threshold of chloride concentration. Concentrations >60 mmol/L indicates CF disease.

Sweat samples were collected from healthy subjects as well as from patients with CF, according to the pilocarpine iontophoresis procedure described above. The samples were split to validate the suitability of the evaluation by the printed chloride sensors, against the gold standard. Before measurement, the sensor was calibrated in NaCl solutions with concentrations from 10^−6^ mol/L up to 1 mol/L. An NaCl solution was chosen along with water. Sweat consists mostly of NaCl, which means that this calibration appropriately mimics the real conditions. After defrosting the samples, each sample was dropped onto the sensor so that both electrodes, the ISE and reference electrode, were completely covered with a continuous sweat film. Using the recorded calibration plot, the concentration of each sweat sample was calculated and is listed in Table 3. According to Gibson et al. [21] and Collie et al. [21,22], a concentration level of chloride above 60 mmol/L indicates a CF disease. As pointed out in Table 3, all samples could be correctly determined and categorized in healthy subjects and CF patients. Figure 4 shows two contrasting samples, healthy (sample 1, 2, and 3) and CF (sample 9), overlaid with the corresponding calibration plot. The dashed line indicates the threshold of 60 mmol/L of chloride.

## 4. Conclusions

In this paper, a screen-printed, low-cost chloride-selective sensor was presented. The sensor showed excellent sensitivity and covered the complete possible concentration range of chloride in sweat. More importantly, the concentration range, which is of high interest for CF diagnosis and the therapeutic monitoring of CF, lies within a linear range, allowing for a good determination of the chloride concentration. As the response time of the screen-printed sensor was only 30 s, fast and simple CF detection and monitoring could be achieved. To further characterize the performance of the sensor, possible disturbance due to interfering ions (e.g., HCO_3_^−^) will be investigated in future work.

To prove proper functionality and the applicability of the chloride sensor for CF diagnosis, measurements of real sweat samples were demonstrated. Comparison against the standard procedure successfully validated a correct distinction between healthy test subjects and patients suffering from CF. With this, the screen-printed sensor could be utilized for the remote monitoring of CF patients, to avoid contact with clinics and therefore to reduce the risk of infections, which is especially useful for maintaining hygiene and distancing regulations in times of a pandemic. Further, as the chloride sensor could be inexpensively manufactured, thanks to screen-printing technology, it can be used as a disposable sensor. In combination with the already published evaluation board for data acquisition and transfer [18], it can be used as a small handheld device. Additionally, due to its flexible design and small dimensions, it is also possible to attach the sensor directly onto the skin. With this, only a low amount of sweat is necessary. The integration of sweat inducement into the sensor system will be investigated in future work. Achieving this would mean that the therapeutic monitoring of CF could be made easy, enabling quick diagnosis and monitoring, with increased comfort for the patients. With this device, the close monitoring of intraindividual changes in sweat chloride levels is possible.

## Figures and Tables

**Table 1:** Membrane compounds of the chloride-selective membrane using the chloride ionophore I –cocktail A with its corresponding portion in parentheses.

Chloride Ionophore I—Cocktail A			Polymer	Solvent
Ionophore	Plasticizer	Anionic Site		
Chloride ionophore I	2-nitrophenyl octyl ether + 1-Decanol	Tridodecylmethyl-ammonium chloride	PVC	1 mL THF on 100 mg compounds
(3.5%)	(65.8%)	(0.7%)	(30.0%)	

**Table 2:** Comparison of screen-printed chloride sensor to the standard sensor system.

Parameters	Screen-Printed Chloride Sensor	Coulometric Titration (FKGO Chloridmeter) ^1^
Detection limit	0.03 mmol/L	10 mmol/L
Response time	<1 min	<1 min
System preparation	5 min	5 min
Sample preparation	-	+
Cost of equipment	<50 €	8200 €
Cost of assay	<3 €	ca. 5 €
Portability	+	-
Usability	+	-

^1^ by Kreienbaum.

**Table 3:** The determined chloride concentration of sweat samples and categorization in health status.

Sample	c(Cl^−^) in mmol/L	Status ^1^ (Healthy or CF)
1	0.9	healthy
2	1.0	healthy
3	1.9	healthy
4	3.0	healthy
5	5.7	healthy
6	0.4	healthy
7	0.6	healthy
8	1.7	healthy
9	129.2	CF
10	100.0	CF
11	63.0	CF

^1^ Derived from different accepted test protocols.
